# Multimodality Imaging of Abnormal Vascular Perfusion and Morphology in Preclinical 9L Gliosarcoma Model

**DOI:** 10.1371/journal.pone.0016621

**Published:** 2011-01-31

**Authors:** Moses M. Darpolor, Robert C. Molthen, Kathleen M. Schmainda

**Affiliations:** 1 Department of Biomedical Engineering, Marquette University, Milwaukee, Wisconsin, United States of America; 2 Department of Medicine, Medical College of Wisconsin, Milwaukee, Wisconsin, United States of America; 3 Department of Biophysics, Medical College of Wisconsin, Milwaukee, Wisconsin, United States of America; 4 Department of Radiology, Medical College of Wisconsin, Milwaukee, Wisconsin, United States of America; University of Illinois at Chicago, United States of America

## Abstract

**Background:**

This study demonstrates that a dynamic susceptibility contrast-magnetic resonance imaging (DSC-MRI) perfusion parameter may indicate vascular abnormality in a brain tumor model and reflects an effect of dexamethasone treatment. In addition, X-ray computed tomography (CT) measurements of vascular tortuosity and tissue markers of vascular morphology were performed to investigate the underpinnings of tumor response to dexamethasone.

**Methodology/Principal Findings:**

One cohort of Fisher 344 rats (N = 13), inoculated intracerebrally with 9L gliosarcoma cells, was treated with dexamethasone (i.p. 3 mg/kg/day) for five consecutive days, and another cohort (N = 11) was treated with equal volume of saline. Longitudinal DSC-MRI studies were performed at the first (baseline), third and fifth day of treatments. Relative cerebral blood volume (rCBV) was significantly reduced on the third day of dexamethasone treatment (0.65±.13) as compared to the fifth day during treatment (1.26±.19, *p<0.05*). In saline treated rats, relative CBV gradually increased during treatment (0.89±.13, 1.00±.21, 1.13±.23) with no significant difference on the third day of treatment (*p>0.05*). In separate serial studies, microfocal X-ray CT of *ex vivo* brain specimens (N = 9) and immunohistochemistry for endothelial cell marker anti-CD31 (N = 8) were performed. Vascular morphology of *ex vivo* rat brains from micro-CT analysis showed hypervascular characteristics in tumors, and both vessel density (41.32±2.34 branches/mm^3^, *p<0.001*) and vessel tortuosity (*p<0.05*) were significantly reduced in tumors of rats treated with dexamethasone compared to saline (74.29±3.51 branches/mm^3^). The vascular architecture of rat brain tissue was examined with anti-CD31 antibody, and dexamethasone treated tumor regions showed reduced vessel area (16.45±1.36 µm^2^) as compared to saline treated tumor regions (30.83±4.31 µm^2^, *p<0.001*) and non-tumor regions (22.80±1.11 µm^2^, *p<0.01*).

**Conclusions/Significance:**

Increased vascular density and tortuosity are culprit to abnormal perfusion, which is transiently reduced during dexamethasone treatment.

## Introduction

Blood vessels have critical role in tumor growth to deliver nutrients and remove wastes from the tumor microenvironment. However, tumor vessels do not adhere to the hierarchical branching pattern of normal vessels but rather form a random branching network with immature vessel wall [1], [2]. As such, the structure and function of the tumor's vascular network is considered abnormal. As a result of abnormal organization of tumor vessels, the blood flow in tumor vessels is abnormal and permeability of vessel is also increased [3], [4].

It has been proposed, with some evidence, that abnormal blood vessels could be pruned by eliminating excess endothelial cells, thereby resulting to a somewhat ‘normal’ vessel state that both functionally and morphologically can provide better delivery of nutrients and therapeutics [2], [5]. Inhibition of VEGF or its receptor leads to apoptosis of endothelial cells and a decrease in vessel diameter, density and permeability [1], [6] and increases oxygen tension [7]. The aforementioned changes could improve the delivery of cytotoxic drugs or accentuate radiation therapy. Also, the loss of VEGF dependence and the subsequent vessel remodeling is marked by coverage of the capillaries by pericytes [8].

Some studies have shown that a combination therapy of antiangiogenic agent and chemotherapeutic [9], [10] or radiation therapy [7], [11], [12], [13] produce additive or synergistic effects as compared to either therapy alone. The hypothesis is that the efficacy of such combined therapy is dependent on a normalization window that is engendered by antiangiogenic agents. From another perspective, this view of vascular normalization seems rather counterintuitive since the normalization of tumor vasculature could potentially augment the growth rate of the tumor. Either way, understanding this process has significant implications for improving therapeutic outcomes. If normalization provides the best time during which treatment should be administered, it is important to know when the normalization occurs, and how to optimize timing, dosing and possibly the combinations of drugs administered. If normalization results in advanced tumor growth, rather than a window of opportunity, treatment strategies must avoid normalization as an endpoint. Most of the recent studies in rodent models have been invasive, involving the measurement of tissue parameters thought to be indicators of vascular maturity. However, non-invasive imaging techniques can be utilized to delineate vascular abnormality in brain tumors. In this regard, it has been demonstrated that DSC-MRI can be used to detect a normalization of perfusion parameters in 9L gliosarcoma tumors treated with either dexamethasone [14] or a specific anti-angiogenic drug [15]. Dexamethasone was shown to have anti-angioenic effects in this rat model [14], [16]. The dexamethasone effect, as previously described [17], desensitizes tumor vessels to VEGF and down-regulates VEGF production from 9L gliosarcoma tumor cells. However, these measurements were performed at just one time point, and not compared to other independent measures of normalization. So, it was not clear whether an inhibition or normalization event took place, and how these changes compare to other independent measures previously used to define normalization.

In this study, we investigated the use of DSC-MRI technique to longitudinally monitor the evolution of the tumor microenvironment before, during, and after dexamethasone treatment with the specific goal of determining the underpinnings of abnormal perfusion. To achieve this goal the MRI measurements of tumor perfusion were compared to quantitative measures of vessel density and tortuosity with microfocal computed X-ray tomography and molecular marker of vascular morphology.

## Results

### DSC-MRI relative CBV measurements

**Figure 1:**
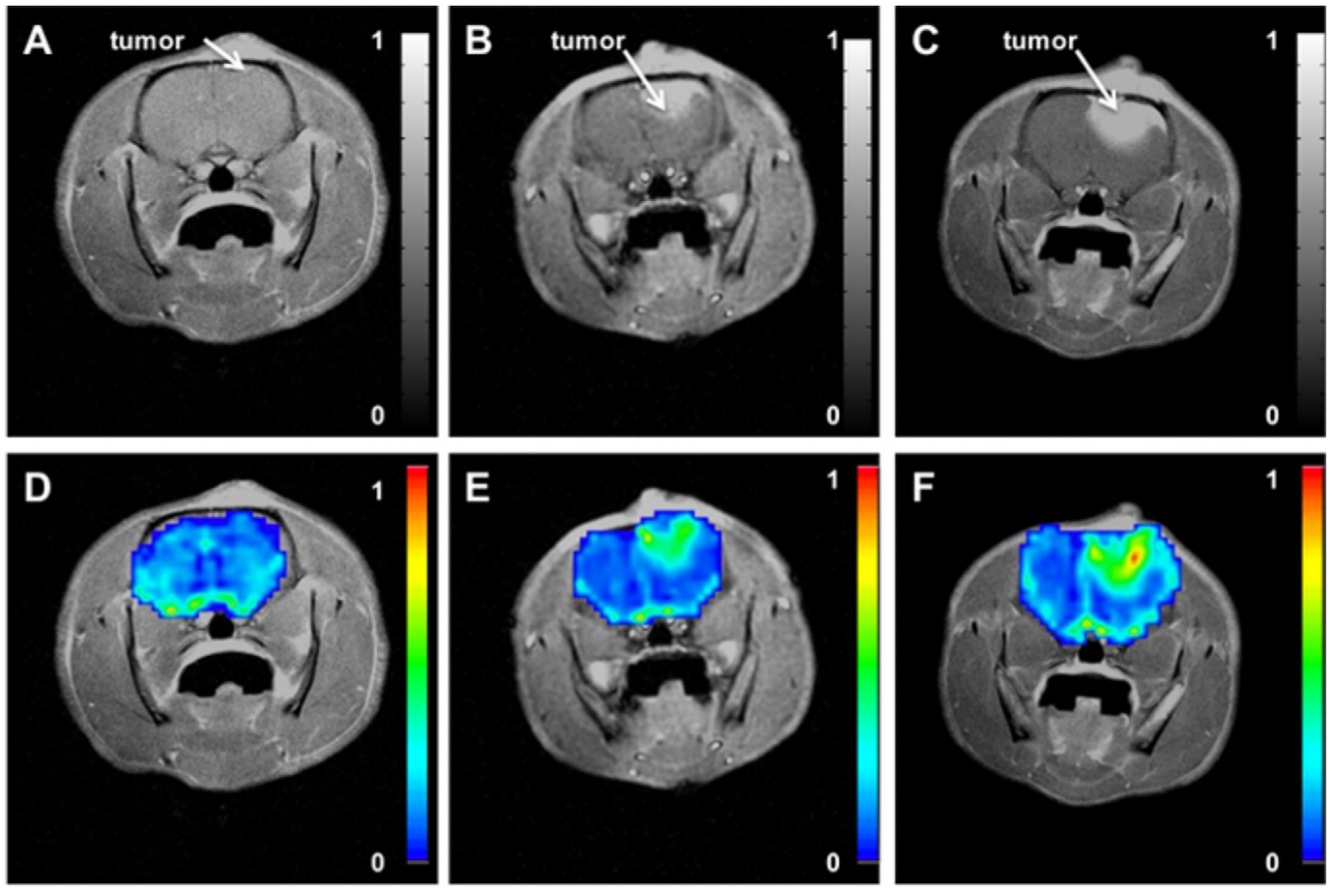
Representative post gadolinium-enhanced T1-weighted MR image of the same rat brain acquired longitudinally on [A] 1^st^ (baseline) day, [B] 3^rd^ day, and [C] 5^th^ day during saline treatment with corresponding parametric relative cerebral blood volume (rCBV) maps on [D] 1^st^ (baseline) day, [E] 3^rd^ day, and [F] 5^th^ day.

**Figure 2:**
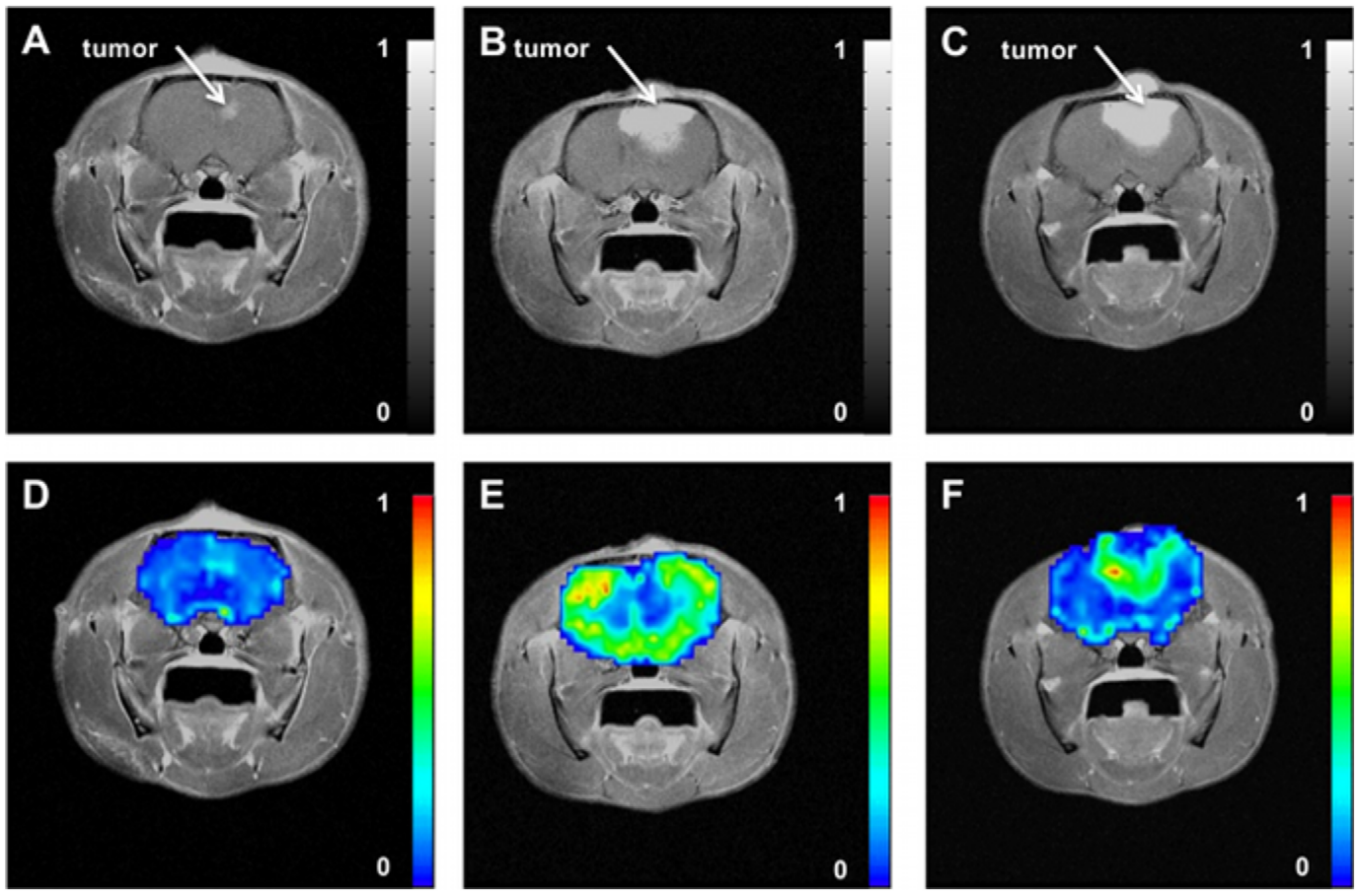
Representative post gadolinium-enhanced T1-weighted MR image of the same rat brain acquired longitudinally on [A] 1^st^ (baseline) day, [B] 3^rd^ day, and [C] 5^th^ day during dexamethasone treatment with corresponding parametric relative cerebral blood volume (rCBV) maps on [D] 1^st^ (baseline) day, [E] 3^rd^ day, and [F] 5^th^ day.

**Figure 3:**
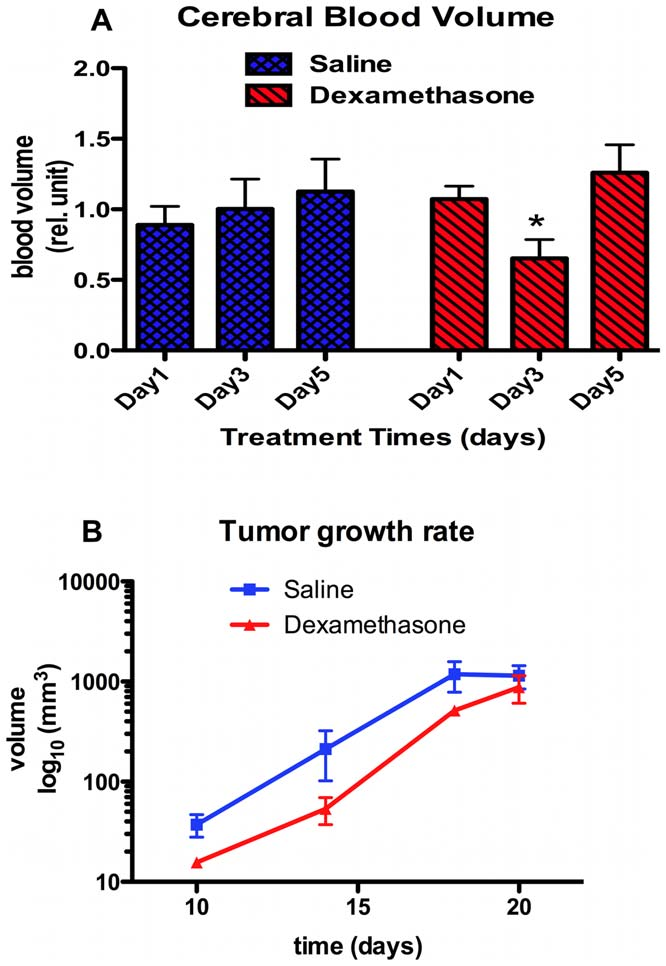
Longitudinal results of [A] quantitative relative cerebral blood volume (rCBV) values of saline treated (N = 11) and dexamethasone treated (N = 13) cohorts *in vivo*, and [B] tumor growth rate. Data points are displayed as mean±SEM, and the p-values were evaluated by one-way ANOVA (^ns^p-value>0.05, *p-value≤0.05, ** p-value≤0.01, *** p-value≤0.001).

Representative parametric color-coded maps from DSC-MRI are masked over the rat brain from saline treated cohort (**Figure 1**) and dexamethasone treated cohort (**Figure 2**) with normalized values ranging from minimum (*blue = 0*) to maximum (*red = 1*). These images were acquired from the same rat at the 1^st^ (baseline), 3^rd^ and 5^th^ day during treatments. In these images, longitudinal changes of tumor blood volume are clearly conspicuous within the same rat. From these maps regions-of-interests were defined in tumor and non-tumor regions for a cohort of rats treated with dexamethasone (N = 13) and another cohort of rats treated with saline (N = 11). The mean CBV value of each tumor region was divided by the mean CBV value of the corresponding non-tumor region. These values are reported as relative cerebral blood volume (**Figure 3A**). One-way analysis of variance indicate a significant difference of means at time points in the dexamethasone treated cohort (*p = 0.02*) whereas no significant difference was observed in the saline treated groups (*p = 0.68*). Relative CBV was significantly reduced on the 3^rd^ day (0.65±.13) of dexamethasone treatment as compared to the 5^th^ day (1.26±.19) during treatment. In saline treated rats, relative CBV gradually increased during treatment (0.89±.13, 1.00±.21, 1.13±.23) with no significant difference on the 3^rd^ day of treatment. The increase of relative CBV in saline-treated rats could be associated with increased abnormal vessels in the tumor, while the transitory drop in these measures may reflect vascular effect in tumor induced by dexamethasone. To validate this claim, separate serial studies were performed utilizing micro-CT and immunohistochemistry to assess the vascular morphology and function of the dexamethasone induced vascular effect. The tumor growth rates of the two cohorts are shown in **Figure 3B**. Tumor doubling time in rat tumors treated with dexamethasone was not substantially longer (2.06±0.34 days, r^2^ = 0.85) than in the saline-treated cohort (3.16±0.22 days, r^2^ = 0.51). Therefore, there is no substantial growth delay of 9L gliosarcoma with dexamethasone treatment.

### Vascular density and tortuosity is reduced during dexamethasone treatment

**Figure 4:**
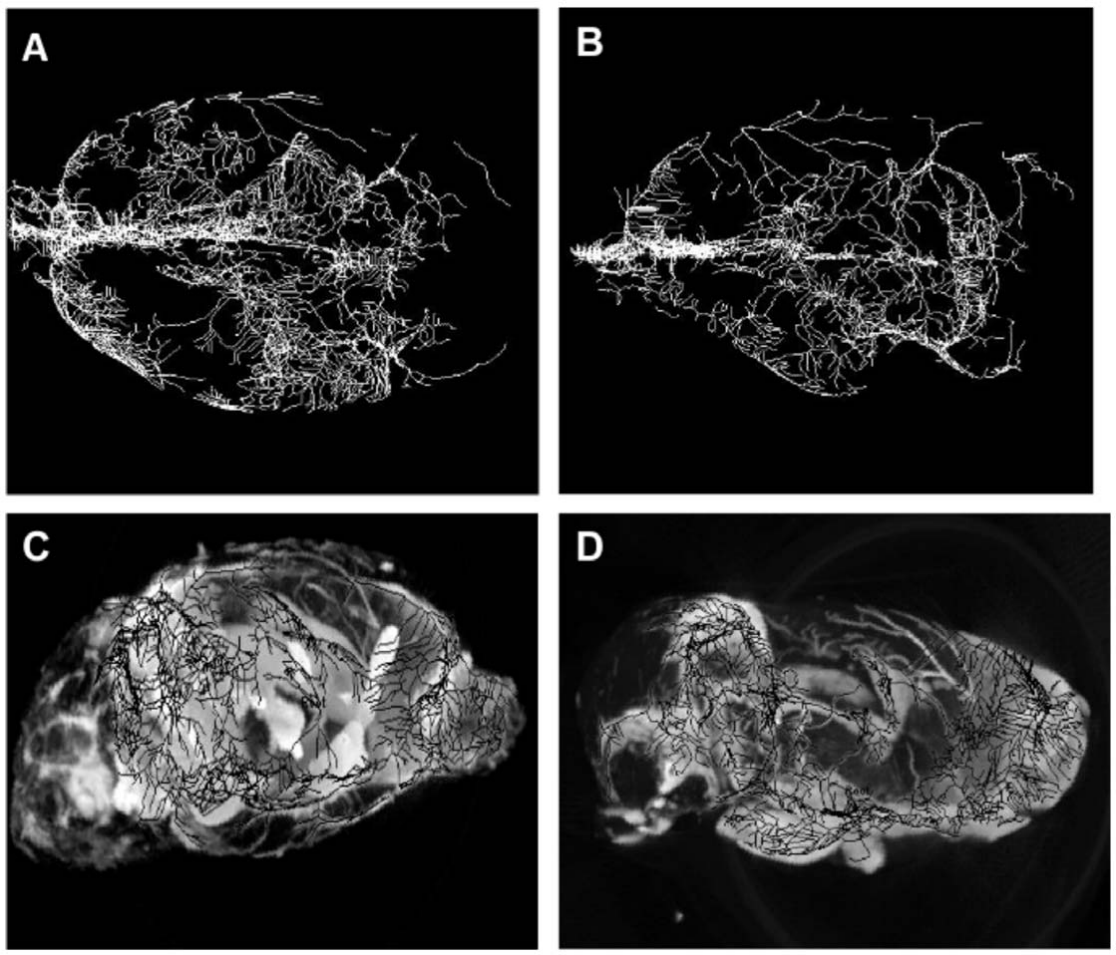
Qualitative morphology from micro-CT data of representative *ex vivo* rat brains. Top view of vessel tree skeleton in [**A**] saline treated rat and [**B**] dexamethasone treated rat. Side view of 3D volume rendered image of [**C**] saline treated rat and [**D**] dexamethasone treated rat.

**Figure 5:**
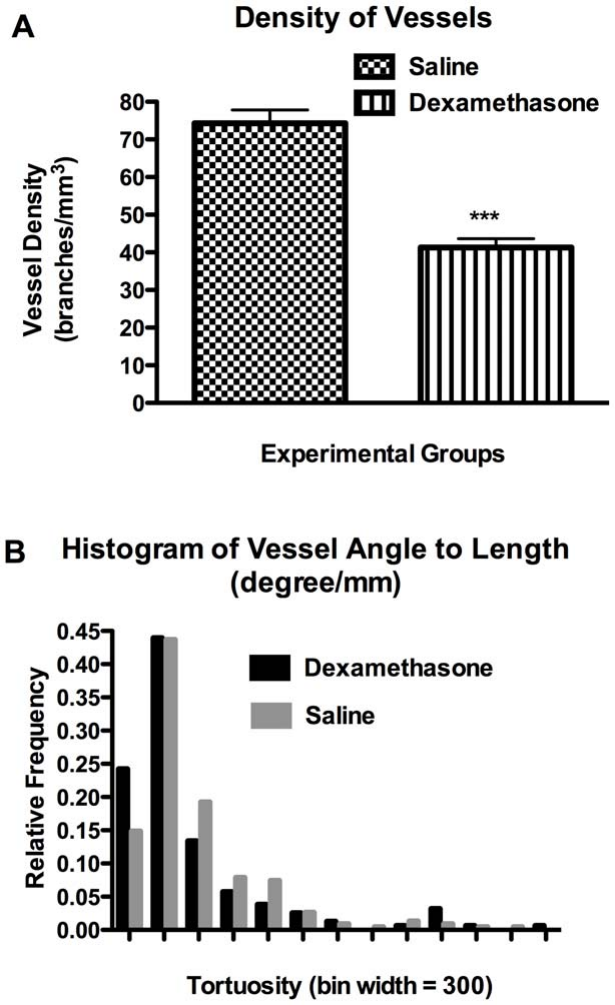
Quantitative morphology from micro-CT data of *ex vivo* rat brains. **[A]** Density of vessel branches in tumor volume of interest for saline treated (N = 4) and dexamethasone treated (N = 5) rats. All bar graphs are displayed as mean±SEM, and the p-value was evaluated by unpaired t-test (*p-value ≤0.05, ** p-value ≤0.01, *** p-value ≤0.001). [**B**] Histogram of tortuosity in saline treated (N = 4) and dexamethasone treated (N = 5) rats. The p-value was evaluated by Mann Whitney U test (p<0.05).

Vascular morphology of *ex vivo* rat brains from micro-CT analysis provides vessel tree skeletons (**Figure 4A-B**) in conjunction with 3D volume rendered X-ray images (**Figure 4C-D**) that were obtained from rat brains treated with either saline or dexamethasone. This tumor model qualitatively exhibits hypervascular characteristics with a substantial increase in the total branched vessels as compared to the same anatomical region in the contralateral hemisphere. Vascular tortuosity was computed from tumor regions with predefined volume-of-interest based on tumor volume. In this context, tortuosity is defined as the branching angle-to-length ratio in units of degree/mm between a daughter vessel and its parent vessel. An increased vessel density (i.e. daughter vessels) and tortuosity were profound in the tumor regions of saline treated rats, as illustrated in **figure 5**. Both vessel density (41.32±2.34 branches/mm^3^, *p<0.001*) and vessel tortuosity (*p<0.05*) were significantly reduced in tumors with dexamethasone treatment as compared to saline treatment (74.29±3.51 branches/mm^3^). This metric may provide an indirect measure of vigorous vessel sprouting and its regression thereof.

### Immunohistochemistry (CD31, PECAM-1) – Morphometric analysis of tumor vessels

**Figure 6:**
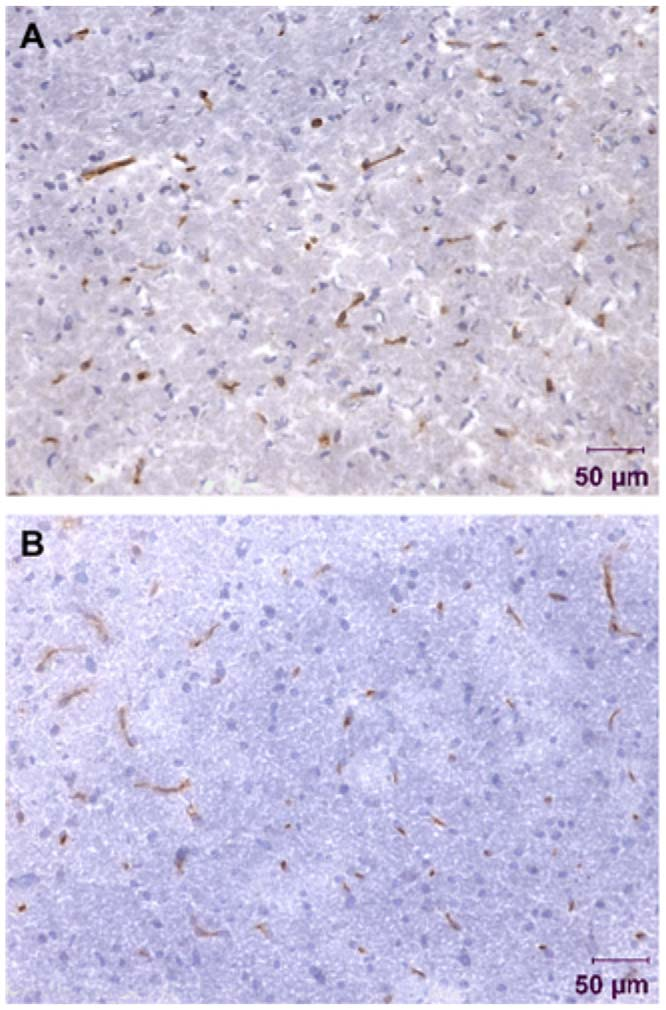
Immunohistochemistry of vessels (anti-CD31, brown) and cell nuclei (hematoxylin, blue) from rat 9L gliosarcoma brain tissue with 0.26 mm^2^ field-of-view at 20× magnification. Tumor regions of [**A**] saline treated and [**B**] dexamethasone treated rats.

**Figure 7:**
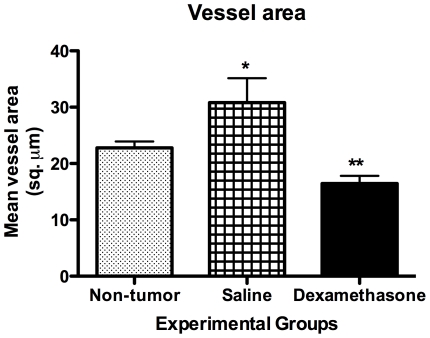
Mean vessel area from anti-CD31 photomicrographs of saline treated (N = 4) rats and dexamethasone treated (N = 4) rats. All bar graphs are displayed as mean±SEM, and the p-values were evaluated by unpaired t-test (*p-value ≤0.05, ** p-value ≤0.01, *** p-value ≤0.001).

We examined the architecture of CD31-immunopositive vessels to assess tumor vasculature, and response to dexamethasone treatment. CD31-immunoreactive vessels were sparse in dexamethasone treated tumors where as vessels in saline treated tumors were more conspicuous with a larger area of CD31-positive cells (**Figure 6A-B**). Our results indicate that dexamethasone treatment caused extensive morphological changes in vessels. In both cohorts, vessels were more localized to dense nuclei as ascertained from the hematoxylin stained image. Tumor vessels areas were quantitatively analyzed on images obtained from the tumor regions. The mean blood vessel counts per field for the dexamethasone treated cohort was 29 whereas the mean vessel count per field for the saline treated cohort was 16. Quantitative analysis (**Figure 7**) of saline treated tumor regions revealed increased vessel area (30.83±4.31 µm^2^, *p<0.01*) as compared to non-tumor regions (22.80±1.11 µm^2^). On the other hand, dexamethasone treated tumor regions showed reduced vessel area (16.45±1.36 µm^2^) as compared to saline treated tumor regions (30.83±4.31 µm^2^, *p<0.001*) and non-tumor regions (22.80±1.11 µm^2^, *p<0.01*) (**Figure 7**).

## Discussion

The present study sought to investigate the underpinnings of abnormal perfusion parametric measures obtained from dynamic susceptibility contrast-magnetic resonance imaging (DSC-MRI) in a rat brain tumor model and to assess vascular changes after dexamethasone treatment. It is demonstrated that parametric measure of DSC, particularly increased relative CBV, reflects vessel density, tortuosity, and molecular tissue marker that define vascular abnormality in tumors. As such DSC-MRI appears to provide a non-invasive method to characterize abnormal vascular perfusion in longitudinal studies.

Imaging techniques that provide information about the microvasculature have become increasingly important in recent years. In part, this is due to the recognition that the growth of tumor tissue is dependent on the development of new blood vessels. Assessment of abnormal vascular perfusion using DSC-MRI techniques was previously investigated in serial or terminal studies [14], [16], [18]. The prospect of dexamethasone to induce change in perfusion parameters of rat brain tumor was indicated at a different time point than the current study. The experimental designs in the previous studies [14], [16] were serial studies in which measurements were made at the end of the study. There were no baseline and intermediate measurements within each rat brain tumor during the treatment. Therefore, only treated group versus untreated group can be compared. Unlike the aforementioned serial studies, we observed in our longitudinal study a significant earlier response to dexamethasone treatment on relative CBV parametric measures, which is transitory.

Although, this study supports the use of DSC-MRI for quantifying abnormal perfusion in rat brain tumors, as it is demonstrated herein, the technique is also sensitive enough to measuring the effect of dexamethasone treatment in the model. This approach offers several practical advantages for integration into other imaging protocols and to facilitate the evaluation of potential antiangiogenic agents in pre-clinical models.

Dexamethasone treatment in the rat 9L brain tumor model can decrease brain tumor-associated vascular permeability (VPF) by two glucocorticoid receptor mechanisms: 1) reduction of the response of vasculature to tumor-derived permeability factors, and 2) reduction of VPF expression by tumor cells [17]. Machein et al demonstrated that dexamethasone downregulates the expression of VEGF/VPF in glioma cells far less efficiently *in vivo* than *in vitro* [19]. They showed *in vitro*, dexamethasone-induced downregulation of VEGF was significantly higher in normoxic (50% in C6 cells and 60% in GS9L cells) than in hypoxic glioma cells (13% in C6 and 30% in GS9L cells). Although there are several possible explanations for the reduced potency of dexamethasone to inhibit VEGF expression *in vivo*, the most likely is that a considerable proportion of tumor cells suffer from hypoxia especially at later stages of tumor growth. Another potential mechanism is the ability of dexamethasone to repair blood-brain barrier dysfunction through the inhibition of cyclooxygenase-2, which also results in prolonged survival [20]. In addition, tumor size and vascular density were reduced in dexamethasone treated rats implanted with 9L gliosarcoma cells [16], [21], [22] and C6 glioma cells [22] after intraperitoneal administration of 3 mg/kg/day. The mechanism by which dexamethasone inhibits tumor growth still remains unclear. Possible mechanisms proposed to account for such an effect are that vessel growth may underly the mechanism by which glucocorticoids decrease brain tumor growth [21] and that tumor cells express glucocorticoid receptors, which makes them the primary target [22].

In collating our findings, it is demonstrated that the vascular density and tortuosity are culprit to abnormal perfusion measurements obtained from longitudinal studies with the utility of DSC-MRI. Parametric measure of this technique, relative CBV, is a potential non-invasive marker for assessing pathological states of vessel attributes in brain tumor.

## Materials and Methods

### Animal Preparation

All procedures with animals were performed in accordance with the recommendations in the guide for the Care and Use of Laboratory Animals of the National Institutes of Health. Our protocol (AUA00000349) was approved by the Institutional Animal Care and Use Committee (IACUC) of the Medical College of Wisconsin (Animal Welfare Assurance No. A3102-01). The 9L cells (MGH, Boston, MA) were cultured and propagated in Dulbecco's modified Eagle's minimum essential medium with 4.5 g/L glucose, 90% 1 mM sodium pyruvate, and 10% fetal bovine serum (GIBCO BRL, Gaithersburg, MD). A total of thirty-four Fisher 344 adult male rats (Sprague Dawley, Harlan, Indianapolis) weighing 250-300 g were anesthetized by an intraperitoneal (i.p.) injection of 0.1 mL/kg of ketamine cocktail made from 1.8 mL of xylazine (20 mg/kg), 3.6 mL of ketamine hydrochloride (200 mg/kg), and 0.6 mL of acepromazine (3 mg/kg). The rat scalps were shaved and wiped with antiseptic swab. Under aseptic conditions, each rat was placed in a stereotaxic head frame (Kopf Instruments, Tujunga, CA), a minor incision was made along the midline, and a 0.15 mm burr hole was drilled at 2 mm to the right of the sagittal suture and 1 mm anterior to the bregma. Approximately 10 µL of medium containing 10^5^ 9L gliosarcoma cells was slowly injected to a depth of 3 mm with a gas-tight syringe (Hamilton, Reno, NV) over a 5-minute period. The 26-gauge needle was slowly withdrawn to prevent extrusion of these cells. Finally, the incision was closed with surgical staple. The animals were returned to their cages and given food and water *ad libitum*. We allowed the tumors to grow up to 14 days post-inoculation before treatment. Tumor volume measurements were obtained from post gadolinium-enhanced T1-weighted MR images. Data from tumor volume measurements were fitted to an exponential growth rate, *y = y_0_×e^(kt)^*, with nonlinear regression implementing least squares method where *y* is the tumor volume (mm^3^), *y_o_* is the measured volume at the start of MRS experiment, *k* is the rate constant, and *t* is the time (day). Tumor doubling time was calculated as 0.69/*k*.

### Dynamic Susceptibility Contrast-Magnetic Resonance (DSC-MR) Imaging

In the longitudinal experiment one cohort (N = 13) was treated (i.p.) with 3 mg/kg per day of dexamethasone sodium phosphate (American Regent Laboratories Inc., Shirley, NY) for five consecutive days, and another cohort (N = 11) was given equal volume of saline. Each rat was anesthetized with injection (i.p.) of the ketamine cocktail previously described and monitored for respiration (60±6 breath/min) and temperature (37±.5°C) during all MRI experiments. All MRI studies were performed using a clinical 3.0 Tesla Signa™ Excite system (GE Healthcare, Waukesha, WI). A quadrature birdcage coil was used for radiofrequency transmission and reception. Echo planar imaging (EPI) was used for acquiring axial DSC images of multiple sections with a temporal resolution of 1020 millisecond. The MR protocol included using a GRE (Gradient Recalled Echo)-EPI sequence (TR: 1 s, TE: 34.5 ms, FOV: 4 cm, Matrix: 64×64, Slice thickness: 2 mm, 0.2 mm spacing, slices = 5). The GRE-EPI data was acquired for a total of 120 seconds before, during and after bolus injection of 2.5 mgFe^+3^/kg monocrystalline iron oxide nanoparticle (MION) (Center for Molecular Imaging Research, Charleston, MA), an intravascular susceptibility contrast agent [23], [24], administered through a permanent-catheter implanted within a femoral vein. MRI experiments were performed on each rat on the 1^st^ (baseline), 3^rd^, and 5^th^ day of treatments.

Programs were developed in-house using Red Hat Linux and Analysis of Functional Neuro-Images (AFNI) software for post-processing of the MRI data. Tracer kinetics analysis was used to compute the perfusion parameters as described previously [14], [15], [25]. Succinctly, cerebral blood volume (CBV) measurements were obtained by integrating the area under the transverse relaxation time curves (i.e. ΔR2*). Next, the interactive AFNI *draw dataset* plugin was used to manually segment the brain from the surrounding tissue, as illustrated in Figure 1A-C, and the color-coded fit parameters were thresholded at Pthr  = 2.6*10^-6^, which corresponds to a Bonferroni corrected α = 0.05. Regions of interest (ROI) were chosen as: untreated tumor, untreated contralateral side, dexamethasone-treated tumor, and dexamethasone-treated contralateral side. Two ROIs were selected in the slice with the largest tumor cross-section. The first ROI was located in the well-delineated contrast enhancing area of the tumor, and the second ROI was located in an area contralateral to the tumor. The mean value of the tumor region was normalized to the mean value of the corresponding contralateral side. Finally, Gadodiamide (0.2 mmol/kg, Omniscan, Nycomed, Princeton, NJ) was injected via the femoral vein to acquire a high-resolution, post-contrast T1-weighted spin echo, image (TE/TR: 12 ms/450 ms; matrix = 256×256; NEX = 16, slices = 5). One-way analysis of variance (ANOVA) was performed on each cohort followed by a Bonferroni post test to compare all pairs of time points at a significance level of alpha = 0.5 (95% confidence intervals). Statistical analysis was performed using GraphPad Prism version 4.0a for Mac OS X (GraphPad Software, San Diego, California USA).

### Microfocal X-ray Computed Tomography Technique

To study the vascular tortuosity of 9L gliosarcoma tumor, microfocal X-ray computed tomography (CT) was used to study nine additional rats, also inoculated with 9L gliosarcoma cells. Of these, five were treated with dexamethasone according to the same protocol described in the DSC section. On the 3^rd^ day of treatments, the time of treatment effect, MRI was performed and brain specimens were further analyzed to validate vessel morphology. For these studies, both carotid arteries were catheterized and infused with a barium sulfate medium (10 ml saline +1 g gelatin +10 g Ba_2_SO_4_) for X-ray contrast. The methods for CT imaging and data analysis have been previously described [26], [27]. Briefly, the CT system is composed of an X-ray source (model Fein-Focus-100.50) with a 3-µm focal spot, a North American Imaging AI-5830-HP image intensifier coupled to a Silicon Mountain Design charged-coupled device camera, and a New England Affiliated specimen micro-manipulator stage mounted on a precision rail, with position information provided by Mitutoyo linear encoders. Image acquisition parameters were as follow: 41 kV, 140 µA, 5° half-cone beam angle, 18.05 cm source-to-object distance, and 78.88 cm source-to-detector distance, 50 frames were averaged for each of the 360 planar acquired at 1° rotational increments. Projection images were carefully preprocessed to compensate for distortions introduced by imaging chain [26], [27]. The Feldkamp [28], [29] cone-beam algorithm was implemented to yield a matrix of 497×497×497 voxels representing a volume of 2.25×2.25×2.25 cm^3^ giving an isotropic resolution of 45.19 µm. Morphometric analysis was performed on the reconstructed images using the Tree module of Analyze 6.1 [30], [31], [32]. The Tree module analysis generates a skeleton of the vessel structure and computes branching angles, segments lengths, and cross-sectional area, which depict the vessel attributes. Volume of interests (VOI) was based on the estimated tumor volume from T1-weighted MR images, and centered at an anatomical landmark in reference to the inoculation coordinates. Vessel density for each VOI was computed as the number of branched vessels to VOI in units of branches/mm^3^. Tortuosity, defined as the branching angle-to-length ratio in units of degrees/mm, was determined for each region. Statistical analysis was performed using GraphPad Prism version 4.0a for Mac OS X (GraphPad Software, San Diego, CA). The Student unpaired t-test and Mann-Whitney test were performed to compare the density and tortuosity of vessels in untreated control rats to dexamethasone-treated rats, respectively.

### Immunohistochemistry (CD31, PECAM-1) – Morphometric analysis of tumor vessels

In a separate serial study (N = 4 saline, N = 4 treated) MRI was performed on the 3^rd^ day of treatment, the time of treatment effect, and the tumor-inoculated rat brain was snap-frozen in liquid isopentane chilled on dry ice, and the brain was overlaid with Tissue-Tek embedding matrix (O.C.T. compound, Sakura Finetek U.S.A, Inc., Torrance, CA) and stored at -80°C. The O.C.T covered brain was sliced into 6 µm sections in a cryostat (Cryocut 1800, Reichert-Jung) cooled to –20°C. Brain sections were fixed in cold acetone (–20°C) for two minutes and rinsed with Tris-buffer saline to remove frozen O.C.T. compound. Sections were incubated with 1.5% H_2_O_2_ in methanol for ten minutes and rinsed twice with TBS. Sections were incubated subsequently with 1% horse serum in TBS. Immunohistochemistry was performed on 6 microns thick slices that were obtained from the extracted rat brains. To examine the vascularity of the tumor regions purified mouse anti-rat CD31 monoclonal antibody (BD PharMingen, San Diego, CA) was used as a primary antibody to stain for platelet endothelial cell adhesion molecule-1 (PECAM-1). Avidin and Biotin blocking kit (VECTOR, Burlingame, CA) solutions were used as recommended by the manufacturer's procedure. All sections were rinsed three times with distilled water for five minutes. These slides were later counterstained with Hematoxylin (Gill's formula, VECTOR, Burlingame, CA) according to the manufacturer's protocol. The histological slices were imaged with 10× and 20× objective lenses at a light microscope, Nikon Model E-400 SPOT Insight Color Camera (Diagnostic Instrument Inc., Sterling Heights, Michigan). These images were further processed in MetaMorph version 6.2 (Universal Imaging Co., Downington, Pennsylvania, PA) to compute the number of vessels and their respective cross sectional area. An unpaired student's t-Test was performed to compare the mean vessel area of untreated control rats and dexamethasone treated rats using GraphPad Prism version 4.0a for Mac OS X (GraphPad Software, San Diego, CA).
